# Multicore-Processor Based Software-Defined Communication/Network Platform for Underwater Internet of Things

**DOI:** 10.3390/s19235168

**Published:** 2019-11-26

**Authors:** Chaohui Luo, Biyun Ma, Fangjiong Chen, Quansheng Guan, Hua Yu, Fei Ji

**Affiliations:** School of Electronics and Information Engineering, South China University of Technology, Guangzhou 510641, China; chhluo@foxmail.com (C.L.); eeqshguan@scut.edu.cn (Q.G.); yuhua@scut.edu.cn (H.Y.); eefeiji@scut.edu.cn (F.J.)

**Keywords:** open architecture, software-defined modems, underwater acoustic communications, underwater wireless networks

## Abstract

Software-defined acoustic modems (SDAMs) for underwater communication and networking have been an important research topic due to their flexibility and programmability. In this paper, we propose a reconfigurable platform for SDAMs based on the TI AM5728 processor, which integrates dual-core ARM Cortex-A15 CPUs and two TI C66x DSP cores. The signal processing and A/D, D/A for physical-layer communication are implemented in the DSP cores. The networking protocols and the application programs are implemented in the ARM cores. The proposed platform has the following characteristics: (1) Due to the high-performance dual-ARM cores, the whole NS3 network simulator can be run in the ARM cores. Network protocols developed in a software simulation platform (e.g., NS3 platform) can be seamlessly migrated to a hardware platform without modification. (2) A new physical-layer module associated with real acoustic channel is developed, such that a data packet generated from the application layer will be transmitted through a real acoustic channel. The results of networking experiments with five nodes are presented to demonstrate the effectiveness of the proposed platform.

## 1. Introduction

Marine resource exploitation is receiving increased attention. Moreover, marine activities have gradually expanded from shallow-water areas to deep ocean areas. Marine resource exploitation is inseparable from the support of underwater communication networks. The Ocean Internet of Things (OIoTs), usually defined as a network of smart, interconnected underwater objects, is considered a promising technology to implement systematic management of miscellaneous marine data [1,2,3]. Due to the fast attenuation of electromagnetic waves, acoustic waves are the most important media of underwater wireless communication [1]. Underwater acoustic communication and networking has become an important research topic in the field of marine information technology. In general, from theoretical research to practical application, underwater acoustic communication technology can be divided into three stages: (1) software simulation, (2) hardware emulation, and (3) experiments and sea tests [2,3]. 

Field tests in marine environments are challenging and costly. In order to improve the reliability of hardware emulation and tests, it is necessary to minimize the workload of secondary development from software simulations to hardware emulation. Therefore, a reconfigurable, software-defined hardware platform is of great significance to marine communication and networking tests. Early reconfigurable underwater acoustic communication platforms mainly focused on the physical layer. These systems are usually composed of a DSP chip (signal processing module) and a FPGA chip (DA/AD module). For example, the reconfigurable modem designed by the Massachusetts Institute of Technology is composed of one motherboard and one daughtercard. It includes four configurable input/output (I/O) channels suitable for multiple-input-multiple-output (MIMO) algorithms and analog anti-aliasing filters implemented by an onboard FPGA and a 300 MHz floating-point DSP with 32 MB SDRAM and 32 MB of flash memory [4]. The daughtercard hosts signal conditioning and amplification stages. The underwater sensor network (UWSN) lab at the University of Connecticut has been actively developing an underwater modem based on MIMO orthogonal frequency-division multiplexing (OFDM). A real-time DSP implementation of both a single-input-single-output (SISO) and a MIMO version of the modem is detailed in [5]. The implementation detailed in [6] demonstrates the real-time capabilities with both a floating-point (TI TMS320 C6713) and a fixed-point (TI TMS320 C6416) DSP, achieving data rates of 3.2 and 6.4 kb/s with a SISO and a 2 × 2 MIMO modem. Readers may refer to [2,3] for more literature about reconfigurable underwater acoustic modems.

Due to the increasing interest in underwater acoustic networking, it is a trend to add the networking protocols in reconfigurable underwater acoustic processing platforms. Traditional DSP chips focus on pipeline signal processing problems, which are not suitable for multithread processing. A general MCU processor has been applied to solve this problem. In [7], an ARM 6410 processor with an external underwater acoustic modem is used to implement the underwater acoustic network protocols. Demirors et al. [8] applied a General-Software-defined Radio Peripheral (USRP) to implement the whole set of communication networking systems from physical layer to network layer. Another common architecture is ARM + DSP dual-core architecture, where the ARM core manages the networking protocols and the peripheral device and the DSP core takes charge of the underwater communication programs [9]. The main disadvantage of the above methods is the poor compatibility between the software simulation and hardware emulation. For example, in a pure software simulation on a network protocol, NS2 and NS3 are the widely used simulation platforms. However, the program of network protocols based on NS2 and NS3 usually cannot be directly applied in the above hardware verification system, and a secondary development, such as code immigration, is required. The University of Padova proposed a simulation platform called SUNSET [10], based on NS2, which is compatible with the hardware verification system, so that the software code can be directly used in the hardware verification system without modification. However, SUNSET considered only the reconfigurable networking protocols. It is interesting to integrate reconfigurable communication and networking. 

A reconfigurable software-defined acoustic modem (SDAM) based on the multicore processor, AM5728 [11], is proposed in this paper. AM5728 is a floating-point, high-performance processor with dual DSP C66x and dual ARM Cortex-A15 clocked at 750 MHz (DSP) and 1.5 GHz (ARM). In our proposed solution, DSP cores host the signal processing for physical layers such as modulation, demodulation, and AD/DA conversion. ARM cores host the networking protocols and extended peripheral drivers (GPS system, remote wireless communication module, etc.), so the platform can run the complete NS3 based on the LINUX system with external memory. NS3 is one of the most common software simulation platforms for networking protocols. Network protocols developed in software simulation platforms (e.g., NS3 platform) can be seamlessly migrated to hardware platforms without modification. Moreover, a new physical-layer module associated with a real acoustic channel is developed, such that a data packet generated from the application layer will be transmitted through a real acoustic channel. The proposed SDAM platform has high integrity and high scalability, and the multiple peripheral devices can be accessed to ARM core to expand the platform functionality.

## 2. Architecture of the Proposed SDAMs 

### 2.1. Hardware Architecture

The proposed hardware platform is composed of a coreboard JN-SOM5728 [12], an interface expansion board JN-OPEN57x [12], a signal-receiving board, and a signal-transmitting board.

**Figure 1:**
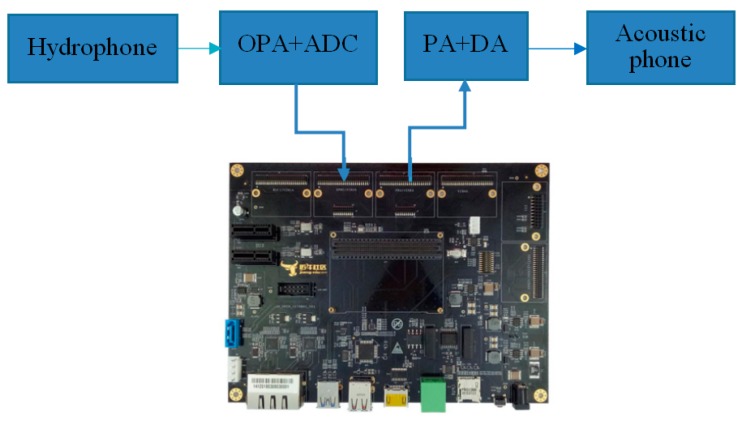
The interface board and the extension modules of an underwater acoustic receiver/transmitter.

The coreboard JN-SOM5728 includes a TI AM5728 CPU and storage resources. JN-OPEN57x is the interface board for the AM57 series, which can be used with coreboard JN-SOM5728. It supports a variety of functional module interfaces, such as dual gigabit Ethernet ports, HDMI, PCIE, SATA, RS232/RS485/CAN, USB2.0/USB3.0, WIFI, GPMC, CSI, and VIN/VOUT. The interface board and the corresponding extension interface modules of the underwater acoustic receiver/transmitter are shown in Figure 1.

**Figure 2:**
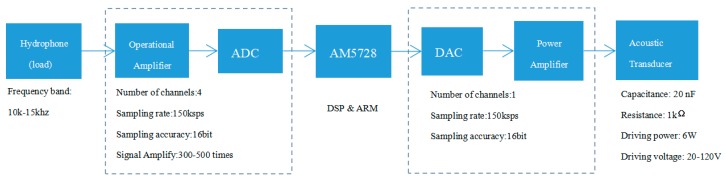
The hardware design block diagram of the receiving/transmitting board for the proposed software-defined acoustic modem (SDAM).

**Figure 3:**
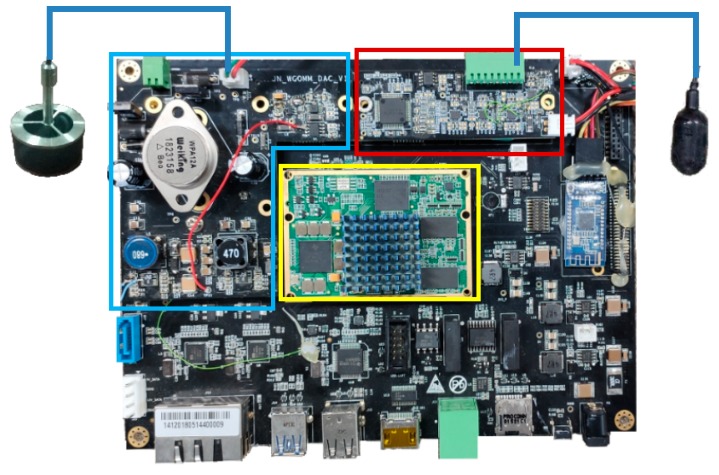
The physical picture of the receiving/transmitting board for the proposed SDAM (yellow— the coreboard, blue—the transmitting board, red—the receiving board).

The receiving board is composed of an operational amplifier circuit and an ADC sampling circuit. The transmitting board is composed of a DAC output circuit and a power amplifier. The hardware design block diagram and some key parameters are shown in Figure 2. The core chips for the receiving/transmitting module are DAC7741 and ADS8588S. The connections between the receiving board, the transmitting board, the coreboard, and the interface board are as shown in Figure 3. The receiving board can be connected to one to four hydrophones, and the transmitting board can be connected to one transducer. The drivers of the receiving board and the transmitting board are implemented in the DSP.

### 2.2. Software Architecture

**Figure 4:**
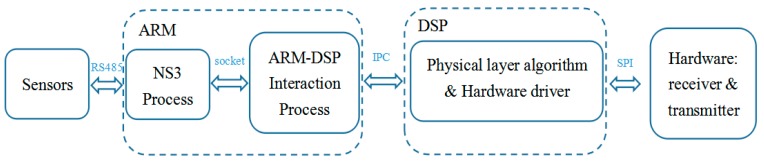
The software architecture of the proposed SDAM modem.

**Figure 5:**
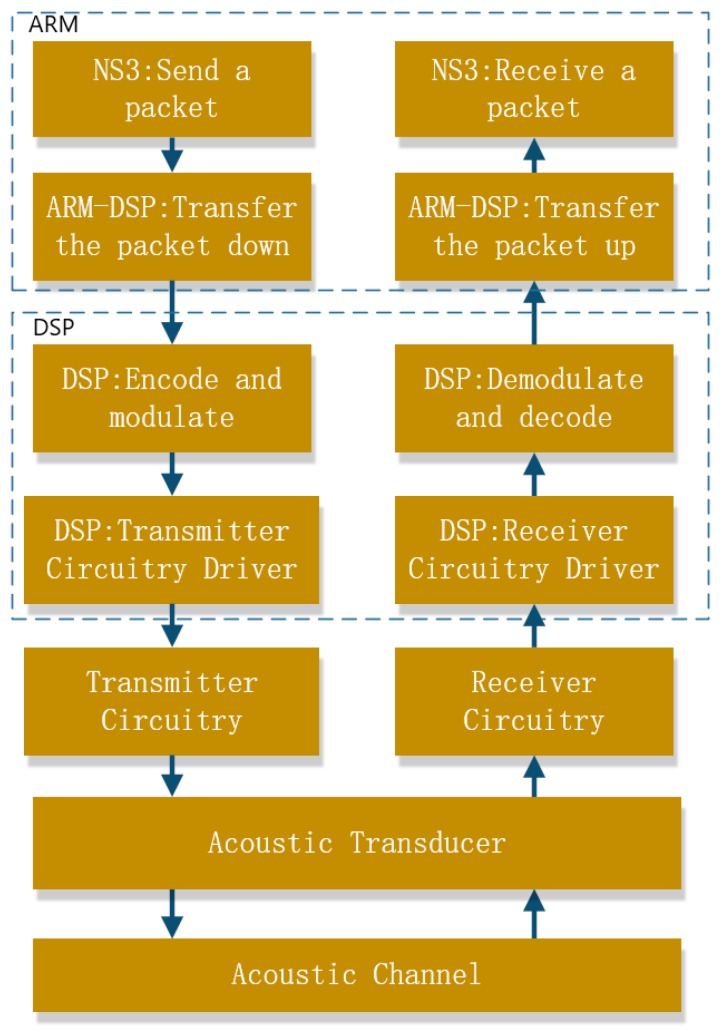
Data flow between different processes and devices.

The software architecture is shown in Figure 4. There are two main processes, the ARM process and the DSP process, which are run in the ARM cores and the DSP cores, respectively. The DSP process responds to the physical-layer algorithms and the driver of the transmitting/receiving hardware. The ARM process responds to the network protocols and the applications. The ARM core and DSP core communicate via the interprocess communication (IPC) scheme provide by TI AM5728. The ARM process can be further divided into two subprocesses—the networking process and the ARM-DSP interaction process. When a data packet is generated by an application and its transmission request is triggered, the packet is sent to the networking process, where the packet header is inserted into the packet. Then, the packet passes through the ARM-DSP process and reaches the DSP process, where the data packet is segmented into frames and standard physical-layer processing, including channel coding. Modulation is applied to each frame to produce an acoustic waveform. The waveform is amplified and sent out by a transducer. The complete flow of packet processing is shown in Figure 5. 

#### 2.2.1. NS3 Networking Process

The NS3 simulator [13] is a widely applied software simulation platform. Due to the high-performance AM5728 processor, we can run a complete NS3 program in a single ARM core. Further, the underwater-acoustic-network (UAN) module [14] developed by Washington University is embedded in NS3 as the basic for protocol development. At first, we port the NS3 simulator over the AM5728 ARM core process, and then the protocol modules of the NS3 and UAN are applied directly to build the stacks on the SDAM. Hence, the proposed SDAM can run the NS3 scripts and use the UAN’s network protocol, including the original built-in protocols or our own designs. 

Generally, in NS3-based software simulations, when a packet transmission request is triggered by an application, no real information bit is generated, and the application layer sends an indication message to the transmission layer. When the packets are delivered layer by layer through the protocol stacks, real information bits are added to the packet header, but the data section remains empty to reduce the memory consumption during the simulation. In the proposed platform, the data packet will be transmitted through a real acoustic channel, hence the data section cannot be empty. In the proposed platform, a new application layer is developed to generate data and fill it into the proper content section of the data packets. 

The real information data for transmission can be randomly generated or collected from external sensors. The interface board provides interfaces to various peripherals such as USB, LAN, PCI, and HDMI; hence, the platform can be connected with various sensors. The processes for sensor data acquisition are embedded in the developed application layer module. Multiple application layer modules are defined to meet different application requirements; for example, the trigger timing of the packet sending in an application layer can be selected to regular trigger or random trigger. 

**Figure 6:**
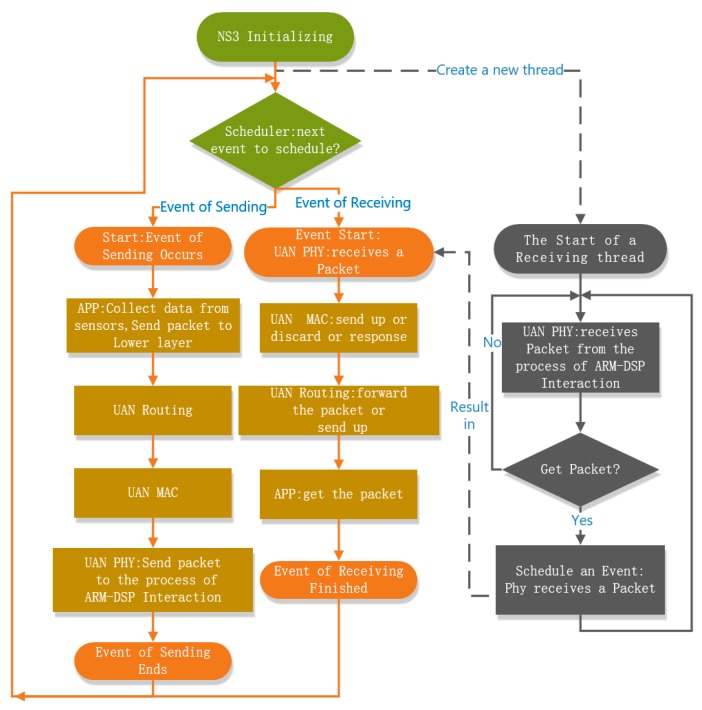
The processing flow of the NS3 process.

Since the whole NS3 simulator can be loaded in the ARM core, the existing protocols in the NS3 platform and UAN module can be directly applied. In the proposed framework, we apply the standard UDP protocol for the transmission layer. In the routing layer, we implement the static routing protocol as outlined in the previous demonstration. In the static routing protocol, the routing table is predefined and the relay node always knows the next hop for transmission. In the UAN modular, without acknowledgement, the Aloha protocol was applied in the MAC layer. In the proposed framework, we extend the Aloha protocol by employing the packet acknowledgement technique. Further, we develop a new physical-layer module in the UAN module and implement bidirectional processing between the NS3 process and the ARM-DSP interaction process. The details of the bidirectional processing are as follows. First, the ARM-DSP interaction process sends the packets received by DSP to the NS3 process via the local socket, and then the UAN physical-layer module generates a receiving event and transfers the packets up to the MAC layer. Second, when data is delivered up to the UAN physical-layer module via the NS3 process, the module sends the packets to the ARM-DSP interaction process via the local socket, which then passes the packets to the DSP core program. A new thread is created to listen to the socket to control the bidirectional data flow. The processing flow of the NS3 protocol process is shown in Figure 6. 

#### 2.2.2. ARM-DSP Interaction Process 

The function of the ARM-DSP interaction process is to enable data transmission between the NS3 process and the DSP process. 

TI’s AM57xx family processors are the ARM application processors built to meet the intense processing requirements of modern embedded products. In an AM57xx processor, different cores in the chip can exchange messages and trigger interrupts with each other via the MAILBOX module. Each core can be configured with the MAILBOX module and read or write messages by DBUS. There are three methods to realize ARM and DSP communication based on the MAILBOX module:

1. The DSP process is called by the OpenCL interface, and the DSP process is considered as an accelerator for the OpenCL interface.
2. The DSP process runs the SYSBIOS system by TI’s RTOS SDK, and then the ARM process and the DSP process communicate via the IPC API message mechanism.
3. The ARM process and the DSP process communicate by the MAILBOX register directly.

The above methods have different efficiencies and complexities. Method 1 has the lowest complexity and highest efficiency, while Method 3 has the highest complexity and the lowest efficiency. In this paper, we apply Method 2, which exhibits satisfactory tradeoff between efficiency and complexity. 

The Message-Q module and the CMEM module provided by TI are employed to realize the ARM and DSP intercore communication via IPC. Message-Q supports transmitting and receiving variable-length, structured messages. It provides APIs for message transmitting and receiving, which are suitable for message exchange between two heterogeneous processing cores. The CMEM module manages one or several consecutive physical block memories and provides address exchanges. In this paper, the Message-Q module is adopted to deliver custom-defined, structured messages such as message type, memory address, and data length. The CMEM module is adopted to address exchange and data copy. The combination of the above two modules more efficiently achieves large amounts of data exchanges between the ARM and DSP cores. 

**Figure 7:**
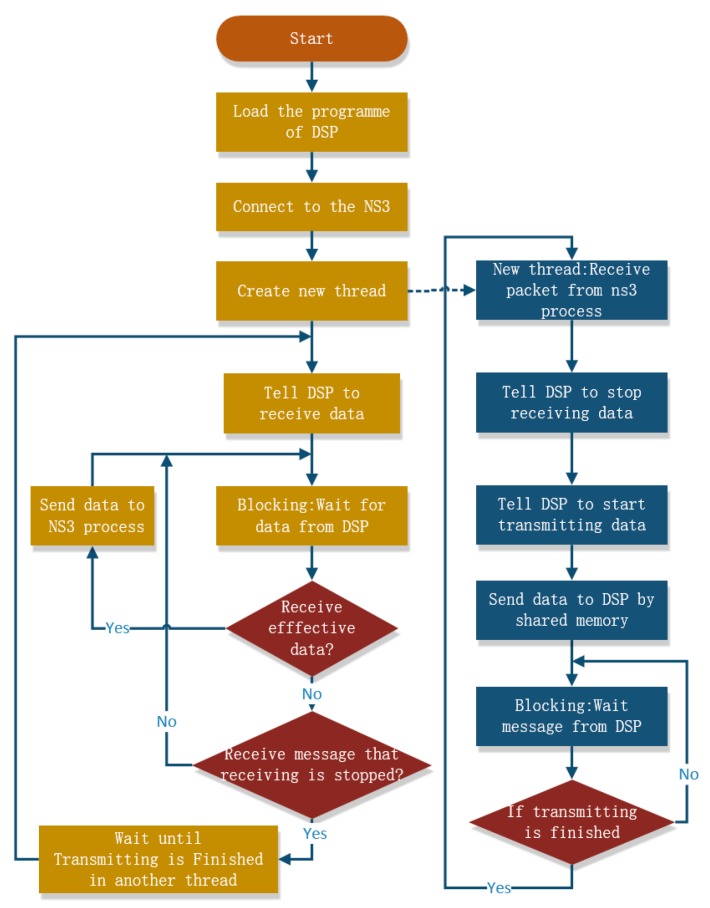
The processing flow of the ARM-DSP interaction process.

To detect the message in Message-Q or socket, there are two methods: blocking or nonblocking. Single-thread, nonblocking polling detection or dual-thread blocking detection can be adopted in the ARM-DSP interaction process to implement the data arrival detection in NS3 or DSP. However, the nonblocking polling methods occupy CPU resources for a long time, so we choose the dual-thread blocking detection method. The processing flow of the ARM-DSP interaction process is shown in Figure 7.

#### 2.2.3. DSP Process

**Figure 8:**
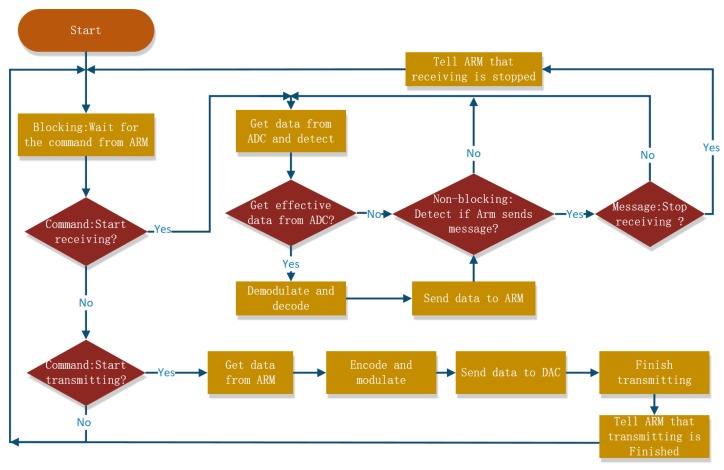
The processing flow of the DSP process.

The processing flow of the DSP process is shown in Figure 8. After initialization, the DSP process waits for the call from the ARM process. According to the demand from the ARM process, the DSP process may call the receiving subprocess or the transmitting subprocess and turn into the receiving or transmitting state. 

The receiving subprocess is a process of cyclically sampling ADC data and receiving ARM messages. The receiving loop ends only when the DSP process receives the receiving end command from the ARM process. The transmitting subprocess is not a loop; it ends the transmitting state after the data is transferred and returns to the main function. At the end of the receiving loop or the transmitting process, a corresponding process end message is sent to the ARM process. 

## 3. Experiments and Validations

### 3.1. Experiments of Physical-Layer Communication

**Figure 9:**
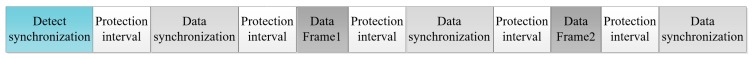
The frame structure (contains two data frames).

MFSK modulation is adopted in the physical layer. Before modulation, the information bits are processed with CRC validation, scrambling, convolutional coding, and interleaving. The data frame structure is shown in Figure 9, where the hyperbolic frequency modulation (HFM) signal with 6 KHz bandwidth and length of 25.6 ms is for synchronization and Doppler compensation. The HFM signal is followed with silence time, forward training symbols, data symbols, and time-reversal training symbols. The parameter list of MFSK modulation is presented in Table 1.

**Figure 10:**
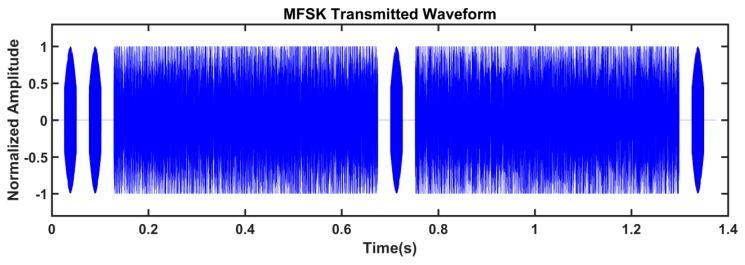
The normalized MFSK transmitted waveform.

**Figure 11:**
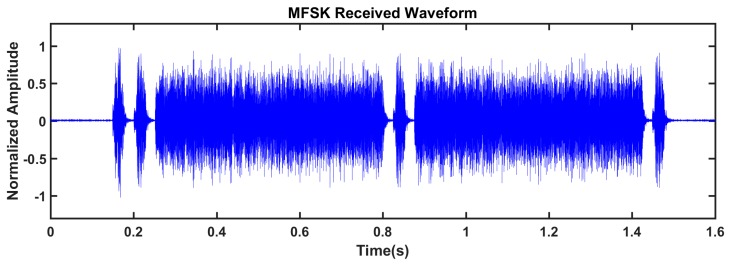
The normalized MFSK received waveform.

The experiments are conducted in an indoor pool. We assemble two nodes, and the distance between the transducer and hydrophone of the two nodes is about 2 m. Node 1 sends the sensor data, and its ARM process transfers the data to the DSP process. After the MFSK modulation, the DSP generates the waveform to be transmitted, as shown in Figure 10. The whole waveform has 110080 points (1.376 s). The waveform contains two frames, and each frame contains 21 bytes of effective information. The received waveform, which is obtained by the receiving circuit in Node 2, is shown in Figure 11. When compared with Figure 10, one observes that the received waveforms in Figure 11 suffer from noise and attenuation. However, the data frames are separable and signal-to-noise ratio is high. After demodulating and decoding, the receiving node can completely recover the transmitted data. 

### 3.2. Networking Experiments

**Figure 12:**
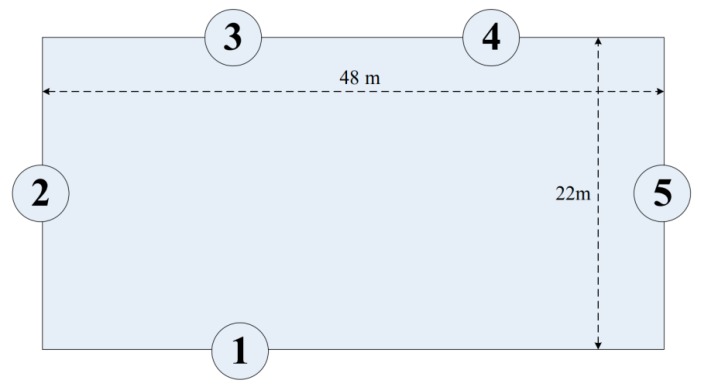
The positions of the five nodes.

To validate the feasibility of the proposed SDAM, we arranged five nodes in a pool 48 m in length and 22 m in width. The positions of the nodes are shown in Figure 12. 

**Figure 13:**
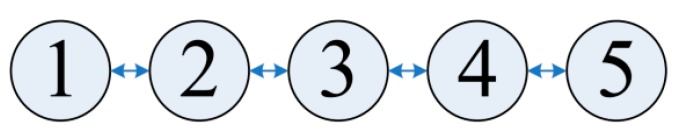
The line topology of the nodes.

The physical layer applied the MFSK modulation as described above, and is implemented in the DSP codes. The MAC layer, the routing layer, and the application layer were configured in NS3 script, and were then run, scheduled, and processed by the NS3 process. The MAC layer adopted the ACK Aloha protocol, and the routing layer applied the static routing protocol. The data link between nodes was set to a line topology, as shown in Figure 13. The triggering time of the packet send event in the application was set to fixed interval time (30 s) or random interval time (0 to 30 s). The destination node was randomly selected to form the other four nodes. The hop address was obtained by the static routing table, and the length of the whole packet was 42 bytes. 

**Figure 14:**
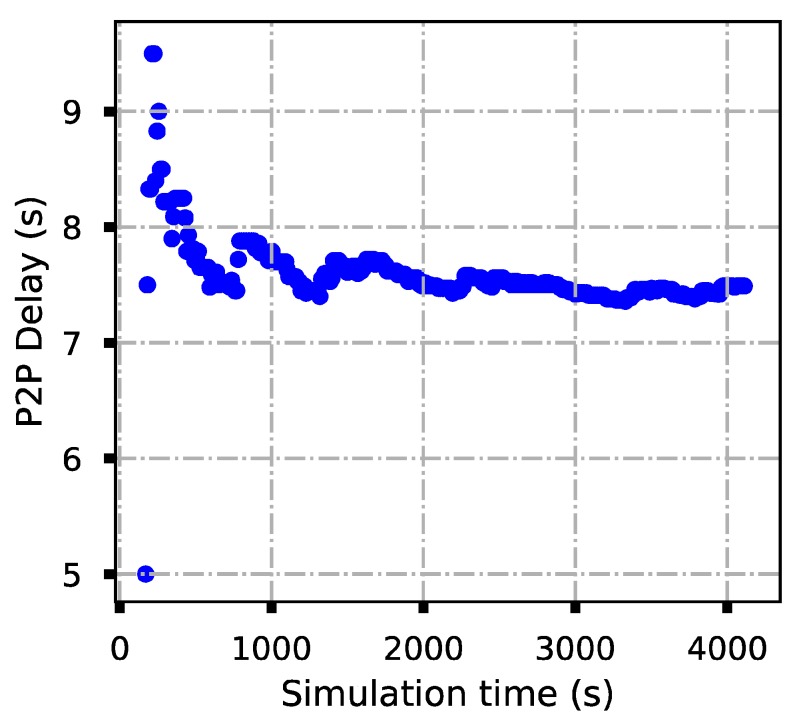
The average delay of point-to-point transmission.

**Figure 15:**
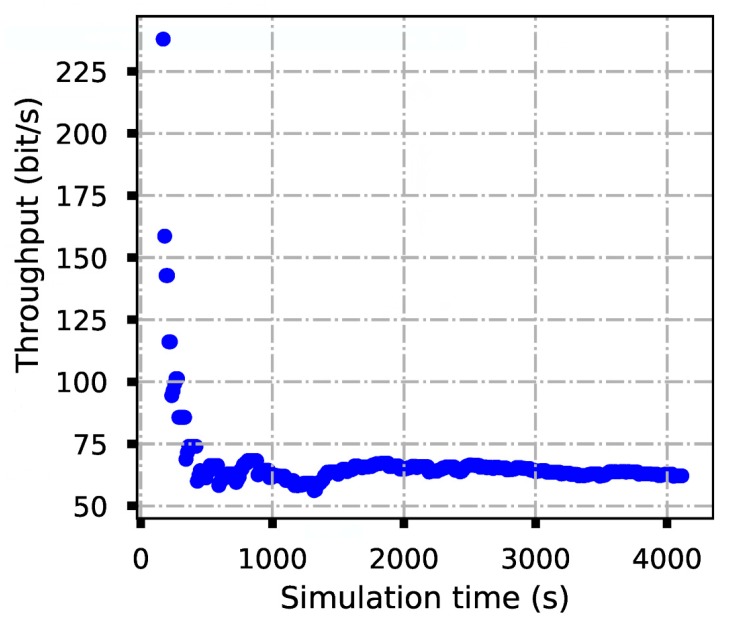
The average throughput.

After about 70 min, we collected the NS3 running logs saved by each node, then extracted the log time and the corresponding packed IDs of the successful sending and receiving packets of each node. Based on the collected information, the system average throughput and the point-to-point average delay were calculated and plotted in Figure 14 and Figure 15, where the delay and throughput were updated every 20 s. From Figure 14 and Figure 15, one observes that at the beginning, the delay and throughput vary rapidly; however, after a period, the average throughput and average delay become stable.

## 4. Conclusions

A reconfigurable SDAM based on the multicore processor AM5728 is proposed in this paper. The DSP cores host the signal processing for physical-layer communication, including modulation, demodulation, and AD/DA conversion. ARM cores host the networking protocols and extended peripheral drivers. The proposed platform can run NS3 with high-performance, dual-core ARM. Additionally, due to the bidirectional interactions between the DSP and the ARM cores, the sensor data of network nodes can be transferred in realistic underwater acoustic channels. We have presented the experimental results of physical-layer communication and networking, which validate the feasibility of the proposed SDAM.

## Figures and Tables

**Table 1:** The parameter list of MFSK communication.

Parameter	Value
Frequency/kHz	9–15
Sampling frequency/kHz	80
Cyclic prefix length/ms	20
Hadamard coding efficiency	1/4
Detect synchronization duration/ms	25.6
Data synchronization signal duration/ms	25.6
Protection interval duration of/ms	25.6
Coded data frame duration/ms	547.2
Peak data rate/bps	258
